# Complete mitochondrial genome of the Endangered long-armed scarab *Cheirotonus gestroi* (Coleoptera: Euchiridae)

**DOI:** 10.1080/23802359.2020.1715888

**Published:** 2020-01-27

**Authors:** Chen Yang, En-Jiao Zhu, Qiu-Ju He, Chuan-Hui Yi, Shao-Ji Hu, Xu-Bo Wang

**Affiliations:** a Key Lab Forest Disaster Warning and Control Yunnan, Southwest Forestry University, Kunming, China;; b Yunnan Academy of Biodiversity, Southwest Forestry University, Kunming, China;; c Yunnan Key Laboratory of International Rivers and Transboundary Eco-Security, Yunnan University, Kunming, China;; d Institute of International Rivers and Eco-security, Yunnan University, Kunming, China

**Keywords:** *Cheirotonus gestroi*, mitochondrial genome, phylogenetic analysis, Endangered insect

## Abstract

The long-armed scarab *Cheirotonus gestroi* is an endangered large insect in southwestern China and neighboring countries. Herein, we present the first complete mitochondrial genome of *C. gestroi*. The 16,899 bp long circular genome consists of 2 ribosomal RNA genes, 22 transfer RNA genes, 13 protein-coding genes, and a non-coding control region. Phylogenetic analysis showed that *C. gestroi* shared the closest evolutionary relationship with *C. jansoni* and that Scarabaeidae and Lucanidae were correctly identified within superfamily Scarabaeoidea. The complete mitogenome sequence will provide a basis for further phylogenetic studies and conservation genetics of the genus *Cheirotonus*.

The long-armed scarab, *Cheirotonus gestroi* (Coleoptera: Euchiridae), is an endangered large insect, mainly living in the mountainous areas of southwestern China and neighboring countries (Yi et al. 2015). The adult individuals of *C. gestroi* possess fascinating metallic color, large body size and disproportionate long arms (mainly males), which make them the top list of insect fans’ collections. In last few decades, the population size of *C. gestroi* has substantially decreased due to illegal collections and habitats fragmentation. The species has been listed as the State Second-Class Protected Animal in China since 1989 (http://www.forestry.gov.cn/main/3951/20180104/1063898.html). Mitogenomics play important roles in conservation biology (Shamblin et al. 2012; Themudo et al. 2015). Herein, we report and characterize the complete mitogenome of *C. gestroi*, which will provide useful genetic evidence for conservation genetics.

In this study, the sample was collected from Huanglian Mountain Nature Reserve (22°53′35″N, 102°11′49″E), Yunnan, China in June 2018 and deposited in the herbarium of Southwest Forestry University (specimen code: BJG_HL2018_032). Genomic DNA was extracted from the muscles of a single adult’s chest using the QIAamp DNA Micro Kit (Qiagen, Hilden, Germany) and then sequenced using Illumina HiSeq X10 (Illumina, San Diego, CA). Genes were assembled by the Assembly by Reduced Complexity (ARC) pipeline (http://ibest.github.io/ARC/) (Hunter et al. 2015), using *C. jansoni.* (NC_023246) as the reference genome (Shao et al. 2014). Protein-coding genes (PCGs), transfer RNA genes (tRNAs) and ribosomal RNA genes (rRNAs) were predicted using the MITOS web server (Bernt et al. 2013).

The complete mitogenome of *C. gestroi* is circular and 16,899 bp in length (Genbank: MN893347). The base composition is 38.8% for A, 31.9% for T, 18.5% for C and 10.7% for G. The genome contains 37 genes, including 13 PCGs, 22 tRNAs, two rRNAs, and a non-coding control region (D-loop). J-strand (majority strand) encodes nine PCGs (NAD2-3, NAD6, COX1-3, CYTB, ATP6, and ATP8), while N-strand (minority strand) encodes four PCGs (NAD1, NAD4, NAD4L, and NAD5). The gene arrangement of *C. gestroi* mitogenome is identical to the most insect mitogenomes (Timmermans and Vogler 2012).

In order to validate the phylogenetic position of *C. gestroi*, the mitogenomes of 15 species in superfamily Scarabaeoidea and the outgroup *Euspilotus scissus* (Coleoptera: Histeridae) were used to construct the maximum likelihood (ML) tree. The ML inference was performed using IQ-TREE (Nguyen et al. 2015) in PhyloSuite (Zhang et al. 2020) with 10,000 ultrafast bootstrap replicates. The result indicates that *C. gestroi* has the closest evolutionary relationship with the same genus species *C. jansoni*. Furthermore, two clades of Scarabaeidae and Lucanidae were correctly identified with high bootstrap values (Figure 1). The mitochondrial genome data of *C. gestroi* present in this study will be beneficial to the further phylogenetic studies of Scarabaeidae and the conservation genetics of the genus *Cheirotonus* species.

**Figure 1.**
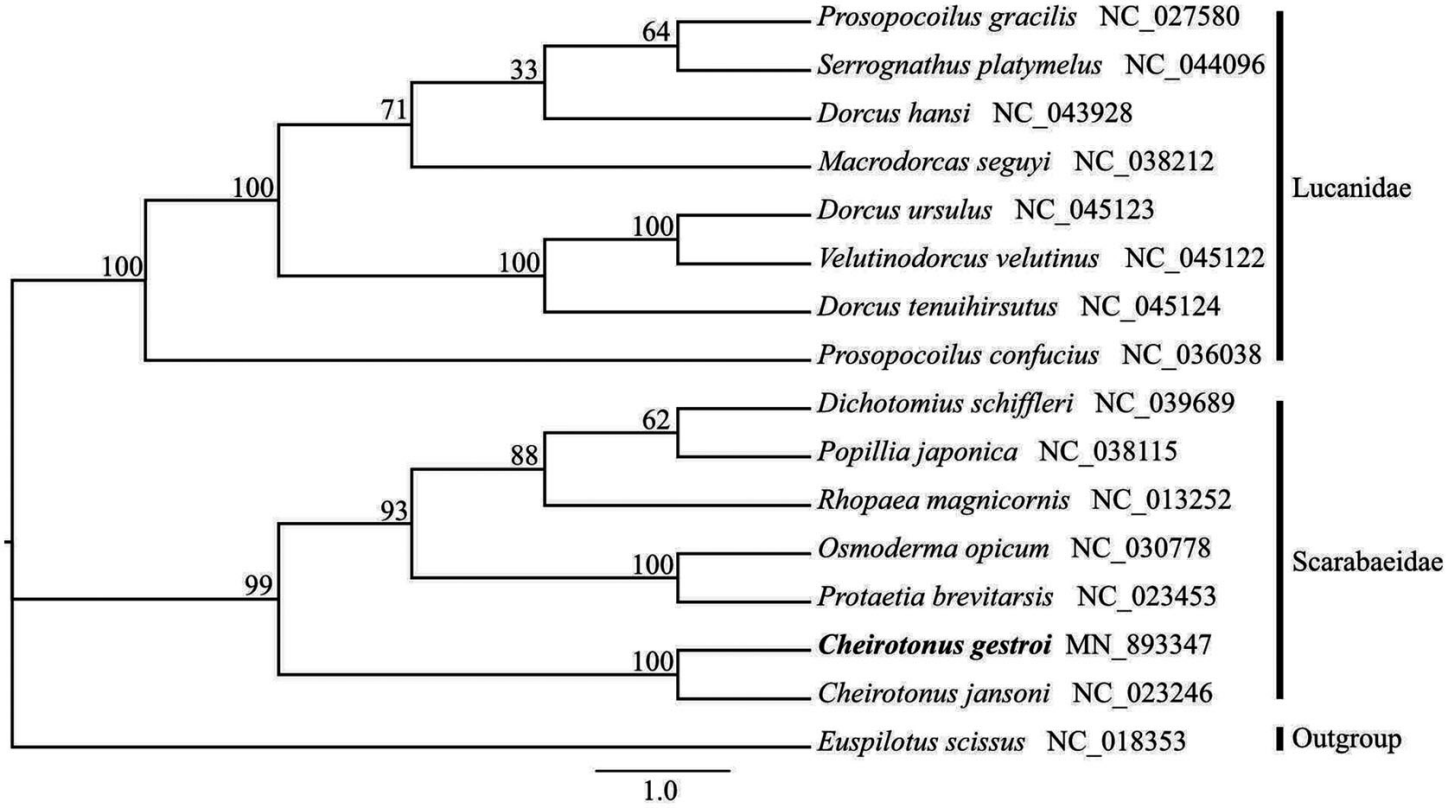
Phylogenetic tree for *Cheirotonus gestroi* and the related species based on concatenated 13 protein-coding genes (PCGs) by Maximum Likelihood (ML) method. Numbers above branches represent Bootstrap support values. The position of *C. gestroi* is marked with bold font.
